# Vibrio cholera Bacteremia Secondary to Ascending Cholangitis in a Patient Not on Chemotherapy or Immunosuppressants

**DOI:** 10.7759/cureus.19853

**Published:** 2021-11-24

**Authors:** Anas A Bogari, Basel M Alsolami, Faisal Al-Husayni, Adeeb Munshi, Maher Alharbi

**Affiliations:** 1 Internal Medicine, National Guard Hospital, Jeddah, SAU; 2 Internal Medicine, King Saud Bin Abdulaziz University for Health Sciences, College of Medicine-Western Region, Jeddah, SAU; 3 Internal Medicine, King Abdullah International Medical Research Center, Jeddah, SAU; 4 Internal Medicine, National Guard Hospital, King Abdulaziz Medical City, Jeddah, SAU; 5 Infection Prevention and Control, National Guard Hospital, King Abdullah International Medical Research Center, Jeddah, SAU

**Keywords:** cholera bacteremia, cholera, ascending cholangitis, bacteremia, vibrio cholera

## Abstract

Non-O1, non-O139 *Vibrio cholera* (NOVC) are considered non-pathogenic organisms, but in some cases, it is known to be responsible for self-limiting intestinal and extra-intestinal infections in immunocompetent individuals. On the other hand, NOVC bacteremia affects mainly immunocompromised patients with significantly high mortality rates. We report a case of an 80-year-old female with a 20-year history of multiple abdominal surgeries. She is also known to have ischemic heart disease and atrial fibrillation. The patient presented with abdominal pain and signs of septic shock. Her abdominal imaging showed features of ascending cholangitis, while her blood culture grew *V. cholera*. She was discharged after completing the course of antibiotics but then came back with a similar presentation. The repeated blood culture showed *Clostridium perfringens*, while other cultures were negative. The patient's condition worsened due to sepsis, and she passed away. NOVC bacteremia is a fatal disease even in hosts who are not receiving immunosuppressants or chemotherapy. It may present without a history of diarrhea or seafood ingestion. In such situations, abdominal imaging is necessary to identify the presence of intra-abdominal infections.

## Introduction

Cholera is one of the most known pandemics in history since it was first observed in India in the year 1817 until the 2020 outbreak in Yemen [1]. *Vibrio cholera* (*V. cholera*) are gram-negative, curved rods like coma with bacterial flagellum used as a hook with hair-like projections called pili helps in bacterial adhesion colonization of intestines subsequently causing the disease [2]. These organisms are intolerant to acidic media but are very resistant to alkaline conditions, they can grow in both aerobic and anaerobic conditions, but they favor the aerobic conditions and thus are called facultatively anaerobic bacteria [2,3].

Over time, cholera pandemics have evolved into two main serogroups, which are O1 and O139 [1,2]. O1 group is the original form since discovering the bacteria, while O139 was identified later in 1992 during the Bangladesh outbreak of Cholera [1,2]. *V. cholera* O1 is divided biologically into two main types, which are classical and El Tor biotype. The main difference between them is that the second type develops a capsule layer around itself, but it produces the same enterotoxins' abilities [4].

On the other hand, some *V. cholera* species are known to be non-pathogenic species and are usually considered colonizers in humans [5]. These species are non-O1 and non-O139 *V. cholera* (NOVC), and they do not have the ability to produce enterotoxins; thus, individuals who carry this type are usually asymptomatic [5]. Nonetheless, it has been reported that NOVC may cause illnesses such as skin and soft tissue infections, meningitis, urinary tract infection, and pneumonia [6]. Bacteremia due to *V. cholera* is seen in immunocompromised patients [7-9].

Herein, we present a case of a patient who is not on immunosuppressants or chemotherapy and found to have *V. cholera* bacteremia secondary to ascending cholangitis.

## Case presentation

We present the case of an 80-year-old female patient known to have ischemic heart disease, heart failure with reduced ejection fraction, and atrial fibrillation. Almost 20 years before her current presentation, the patient had a history of ascending cholangitis post endoscopic retrograde cholangiopancreatography, complicated by an iatrogenic esophageal perforation requiring surgical repair and cholecystectomy. In 2015, the patient had another abdominal perforation secondary to an ischemic bowel due to arterial emboli. 

The patient presented with a one-week history of abdominal pain, dysuria, and fever. Her vitals showed a blood pressure of 86/55 mmHg and a heart rate of 127 beats per minute, which recovered after 500 mL of normal saline. Her urine dipstick revealed 100 leukoestrase and positive nitrites. Basic blood investigations were normal, and cultures were sent. She was started on Ceftriaxone 1 g once a day to treat her urinary tract infection. The cultures came back negative apart from blood cultures which grew *V. cholera* within a day of incubation (Table 1 shows the antibiotics' sensitivity).

**Table 1:** Antibiotic sensitivity to Vibrio cholera in the blood culture. MIC, minimum inhibitory concentration.

Antibiotics	Sensitivity	MIC
Amoxicillin/Clavulanate	Sensitive	4
Ampicillin	Sensitive	≤2
Ceftazidime	Sensitive	≤1
Ciprofloxacin	Sensitive	≤0.25
Gentamicin	Sensitive	≤1

**Figure 1:**
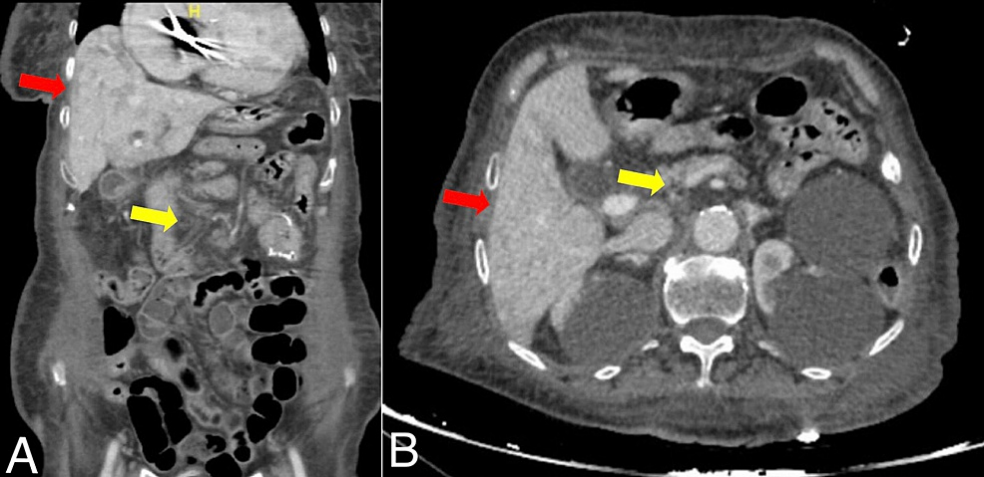
(A) Coronal and (B) sagittal views of the patient’s abdominal computed tomography revealing an enhancement of the liver (red arrows) and common bile duct dilatation (yellow arrows).

The patient was clinically improving but to identify the bacteremia source, a stool culture was sent, and abdominal computed tomography (CT) was arranged. The stool culture was negative, and the CT showed a common bile duct dilatation with a heterogeneous enhancement of the liver, representing cholangitis, but no obstruction was observed (Figure 1).

Since the patient was improving and her CT findings were related to her previous surgeries, no intervention was done. 

She completed a seven-day course of Ceftriaxone, and repeated blood culture did not grow any organism. She was discharged home, but a few days later, she returned with severe sepsis without a clear focus. Cultures were sent, and the results showed *Clostridium perfringens* bacteremia. She was started on an intravenous metronidazole loading dose of 750 mg, and then she was kept on 375 mg every 6 hours as a maintenance dose. The patient's condition worsened as her level of consciousness and blood pressure dropped, requiring norepinephrine infusion and intubation. Her intensive care unit stay was complicated with ventilator-associated pneumonia. After two days, the patient's condition deteriorated, and she passed away.

## Discussion

*V. cholera* bacteria are categorized into more than 200 serotypes, with O1 and O139 being the main serogroups, and they are known to produce toxins and cause classical cholera that typically has a clinical presentation of diarrhea. Furthermore, the NOVC serogroups are often non-epidemic strains, and they are reported as sporadic cases [10]. NOVC may also cause life-threatening infections like bacteremia, mainly in immunocompromised patients like those with cirrhotic liver diseases, with cancer, on immunosuppressive therapy, and post-splenectomy [10]. In our case report, our patient is a healthy individual with normal immunity as she is not a cancer patient, not known to have human immunodeficiency virus infection, and not on any immunosuppressant medications. She is also not a known diabetic or have liver cirrhosis. The case at our hand was not associated with seafood consumption, so sporadic gastroenteritis and life-threatening infections following the consumption of raw seafood or exposure of damaged skin to contaminated saltwater were excluded [10,11]. Furthermore, watery diarrhea, although bloody or mucoid patterns are possible, and abdominal pain are the most common clinical symptoms of NOVC [12]. Other presentations are considered less common, such as hepatic infections, cerebral and peritoneal abscesses, pneumonia, peritonitis, skin infection, cellulitis, and cholecystitis [12]. Nevertheless, our patient’s history is significant for multiple abdominal surgeries, including biliary tree procedures, which raise the possibility of colonization in the biliary tree mainly and intestinal wall. She had a history of bacteremia with two different organisms that can grow in the intestinal wall as normal flora. *V. cholera* serogroup identification could not be made for our patient due to the unavailability of antiserum identifying enterotoxins [13]. However, our patient’s CT findings and the second bacteremia episode due to *Clostridium perfringens* made her cholangitis the most likely cause as other cultures did not grow either organism. Moreover, given the natural history from previous studies, we presume that NOVC serogroups are the cause of these events, which raise the possibility that multiple intraabdominal surgeries can alter the normal flora bacteria and can cause different colonization of the regular normal flora, especially in an area that is endemic with NOVC like Saudi Arabia [14]. In addition, a review conducted by Chen et al. to study a series of 83 NOVC infections in Taiwan found that 45 of these cases had acute gastroenteritis (54.2%), where 12 experienced a biliary tract infection (14.5%) and 11 suffered from primary bacteremia (13.3%) [15]. Besides, adult septicemia occurs predominantly in patients with underlying liver cirrhosis, immunodeficiency, hematological malignancies, diabetes, AIDS, or lymphoma-related conditions [10]. Deshayer et al. reported 347 cases of *V. cholera* bacteremia, and in 96% of these cases, the patients have associated conditions [16]. The most common associated disorders were liver cirrhosis (55%) followed by malignancy (20%). However, the source of infection was only identified in 87 of these patients. Unlike classical cholera, antimicrobial therapy is essential for the management of extraintestinal infections with *V. cholera*. However, antimicrobial susceptibility testing is essential because of the lack of standard clinical guidelines for these infections [12]. 

## Conclusions

*V. cholera* can be found as normal flora in the intestinal wall and biliary tree and can cause serious infections even in individuals who are not receiving chemotherapy or immunosuppressants. Multiple abdominal surgeries, including biliary tree procedures, are significant in our patient's history, raising the risk of colonization in the biliary system and intestinal wall. Detecting *V. cholera* bacteremia warrants abdominal imaging to rule out intra-abdominal infections, as the risk of colonization may increase with multiple intra-abdominal surgeries.

## References

[1] (2021). Cholera. https://www.who.int/news-room/fact-sheets/detail/cholera.

[2] (2021). Diagnosis and Detection | Cholera | CDC. https://www.cdc.gov/cholera/diagnosis.html.

[3] Nakasone N, Iwanaga M (1990). Pili of Vibrio cholerae non-O1. Infect Immun.

[4] Calia KE, Murtagh M, Ferraro MJ, Calderwood SB (1994). Comparison of Vibrio cholerae O139 with V. cholerae O1 classical and El Tor biotypes. Infect Immun.

[5] Gyobu Y, Kodama H, Sato S (1991). [Studies on the enteropathogenic mechanism of non-O1 Vibrio cholerae. II. Lethality, adhesion, colonization and cytopathogenicity of enteropathogenic strains]. Kansenshogaku Zasshi.

[6] Chowdhury G, Joshi S, Bhattacharya S (2016). Extraintestinal infections caused by non-toxigenic Vibrio cholerae non-O1/non-O139. Front Microbiol.

[7] Issa H, Shorman M, Bseiso B, Al-Salem AH (2009). A case of O1 Vibrio cholera bacteremia and primary peritonitis in a patient with liver cirrhosis. Gastroenterol Res.

[8] Wei Y, Yu Y, Yang H (2018). Non-O1/Non-O139 Vibrio cholera bacteremia in a patient with autoimmune liver disease. J Emerg Crit Care Med.

[9] Xu TH, Forrest GN (2021). Non-toxigenic Vibrio cholerae in an autologous stem cell and renal transplant recipient. Transpl Infect Dis.

[10] Feghali R, Adib S (2011). Two cases of Vibrio cholerae non-O1/non-O139 septicaemia with favourable outcome in Lebanon. EMHJ.

[11] Bonnin-Jusserand M, Copin S, Le Bris C (2019). Vibrio species involved in seafood-borne outbreaks (Vibrio cholerae, V. parahaemolyticus and V. vulnificus): review of microbiological versus recent molecular detection methods in seafood products. Crit Rev Food Sci Nutr.

[12] Kaki R, El-Hossary D, Jiman-Fatani A, Al-Ghamdi R (2017). Non-O1/non-O139 Vibrio cholerae septicaemia in a Saudi man: a case report. JMM Case Rep.

[13] Finkelstein RA (1996). Cholera, Vibrio cholerae O1 and O139, and other pathogenic Vibrios. Medical Microbiology.

[14] Davis CP (1996). Normal flora. Medical Microbiology.

[15] Chen YT, Tang HJ, Chao CM, Lai CC (2015). Clinical manifestations of non-O1 Vibrio cholerae infections. PLoS One.

[16] Deshayes S, Daurel C, Cattoir V, Parienti JJ, Quilici ML, de La Blanchardière A (2015). Non-O1, non-O139 Vibrio cholerae bacteraemia: case report and literature review. Springerplus.

